# Supplementary material for: Scaling laws in natural conversations among elderly people
Source: PLoS One. 2021 Feb 19;16(2):e0246884. doi: 10.1371/journal.pone.0246884 (PMC7894956; doi:10.1371/journal.pone.0246884)
Supplement: S1 File — (PDF) [file pone.0246884.s002.pdf]

Supporting information for
"Scaling laws in natural conversations among elderly people"

Masato S. Abe[1] and Mihoko Otake-Matsuura[1]

[1]RIKEN Center for Advanced Intelligence Project, Nihonbashi 1-chome Mitsui Building, 15th floor, 1-4-1 Nihonbashi, Chuo-ku, Tokyo 103-0027, Japan

Tel.: +81-048-467-3627

S1. Basic characteristics of the analysis of words

In our study, we recorded conversations among healthy elderly people, and the length of the total recordings was approximately 7 hours for each of the 16 groups. There was no break in conversations in any of the data. Therefore, we obtained recordings of approximately 1.75 hours from each participant; each group had four participants, except for one group that had five participants. However, there were variations among participants in the number of words spoken. The distributions of words and different words are shown in S1 Fig. Some participants were talkative, while others were not. The main results for the association between cognitive scores and the scaling exponent (Fig 2) was obtained from all data sets.

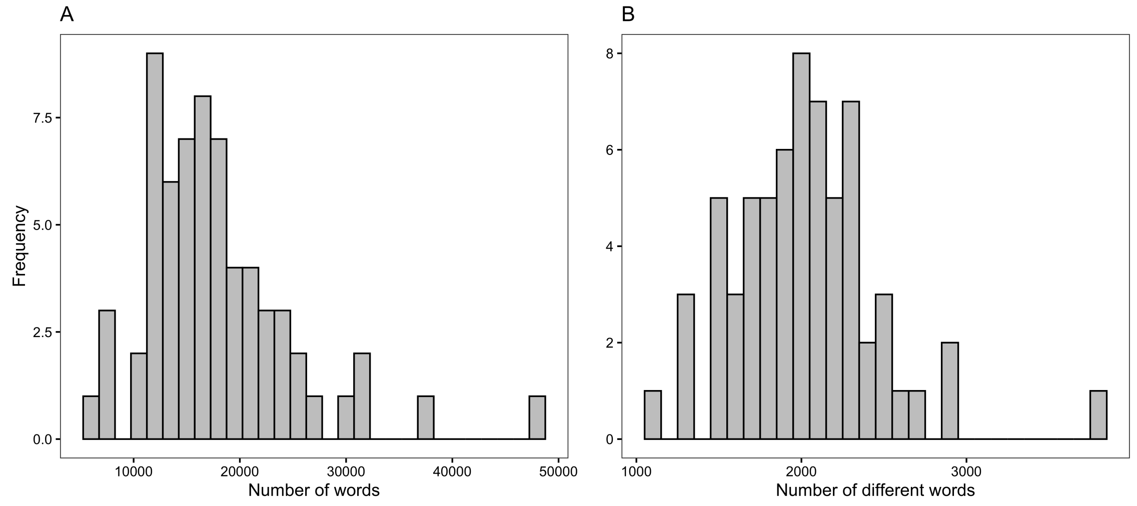

S1 Fig. Distributions of the number of words and different words

(A) Number of words spoken. (B) Number of different words. Sample size, n = 65.

S2. Fitting procedure for Zipf distribution

We used rigorous statistical methods to fit a probability distribution to the empirical rank-word frequency relationship [1,2]. Here, the candidate distributions included a power-law distribution, a shifted power-law distribution, a power-law with exponential cut-off (tail), a log-normal distribution, a Weibul distribution, and a double power-law distribution. The probability mass functions for such distributions are shown in S1 Table. We estimated the parameters of each distribution numerically by maximizing the log-likelihood using the Nelder-Mead method implemented in the optim function of R. The constant C is the value for normalizing, which was obtained as follows:

$$\sum_{r=1}^{N_{\max}} F(r;\, U) = 1$$

where U is a set of parameters, and $N_{\max}$ is the maximum number of different words for each participant.

S1 Table. Probability mass functions for each fitting model

Distribution	Probability mass function $F(r;\, U)$
Power-law	$Cr^{-\alpha}$
Shifted power-law	$C(r+b)^{-\alpha}$
Power-law with exponential cut-off (tail)	$C\exp(-br)r^{-\alpha}$
Log-normal	$Cr^{-1}\exp(-0.5(\ln r-\mu)^2/\sigma^2)$
Weibull	$Cr^{\alpha-1}\exp(-br^{-\alpha})$
Double power-law	$C\begin{cases} r^{-1}, & (r \le b) \\ b^{\alpha-1}r^{-\alpha} & (r > b) \end{cases}$

After estimating the parameters using maximum likelihood estimation (MLE), we calculated the Akaike Information Criteria (AIC), which is defined as

$\mathrm{AIC}_i = -2$ (log-likelihood of model i) + 2 (number of parameters in model i)

Then, to compare these distributions in terms of model selection, the Akaike weights = w_i of the fitting model i was calculated following[3]:

$$w_i = \frac{\exp\left[-(\mathrm{AIC}_i - \mathrm{AIC}_{\min})/2\right]}{\sum_{j=1}^{R}\left\{\exp\left[-(\mathrm{AIC}_j - \mathrm{AIC}_{\min})/2\right]\right\}},$$

where R is the number of candidate models (6), and $\mathrm{AIC}_{\mathrm{min}}$ is the minimum AIC in the fitting models. The value ranges from 0 to 1 and represents the probability of the model given the data [3]. Therefore, the model producing a w_i close to 1 provides supporting evidence for the model. S2 Fig illustrates an example of the fitting result of a particular participant.

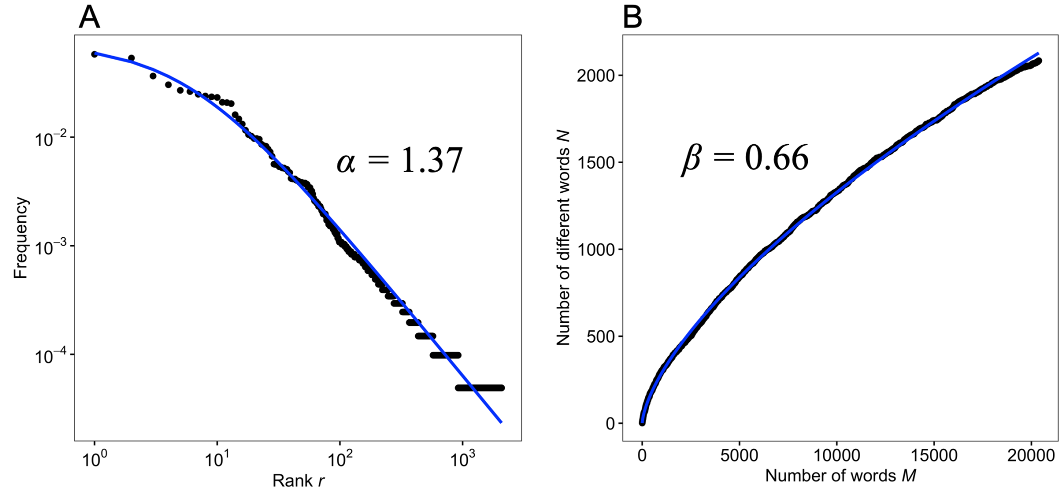

S2 Fig. Examples of fitted distribution and function to data

The black dots represent the empirical data for a participant, and the blue lines represent the fitted shifted power-law distribution for the rank-frequency relationship (A), and for the relationship between the number of words and distinct words (B).

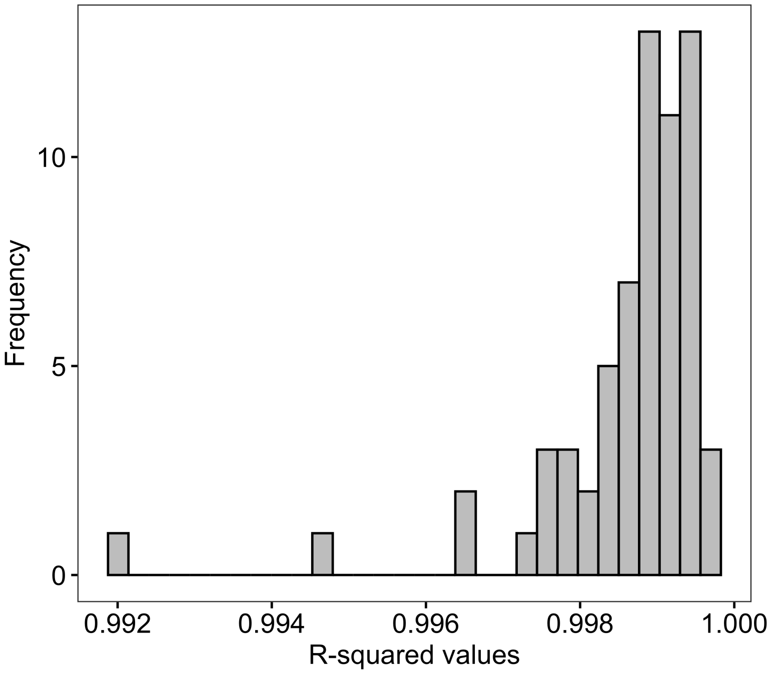

S3 Fig. Distribution of R-squared values for fitting Heaps' law

R-squared values were calculated for the model with the best fitted parameters.

S3. Results for data without prefixes

We analyzed words data without prefixes because each participant may have a different prefix length. The results were quite similar to the main results as shown in S4 – S8 Figs. and S2– S3 Tables.

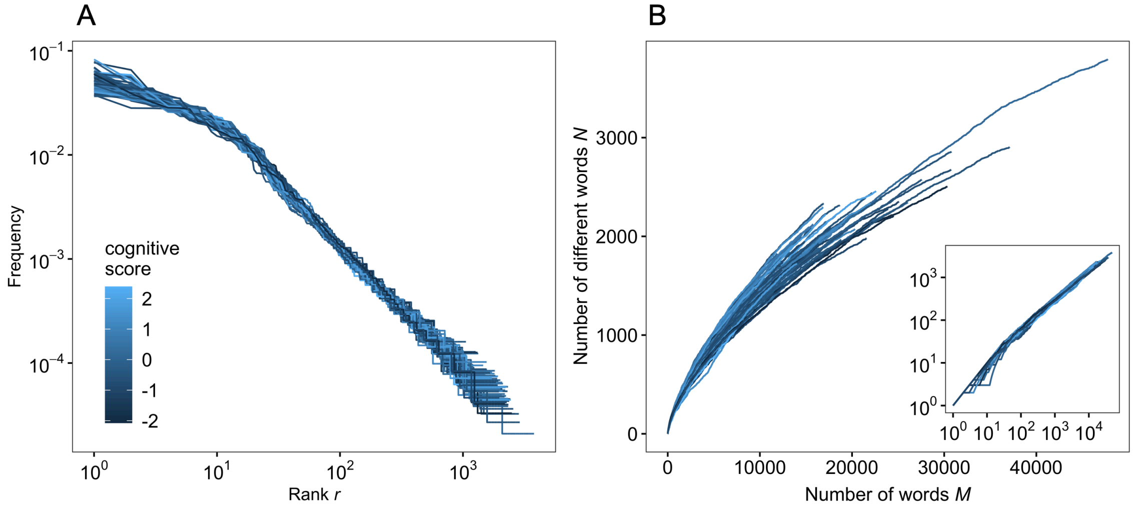

S4 Fig. Zipf's law and Heaps' law in natural conversations of elderly people in the case of no prefixes.

The prefixes were removed from words data. Otherwise, same as Fig. 1 in the main text.

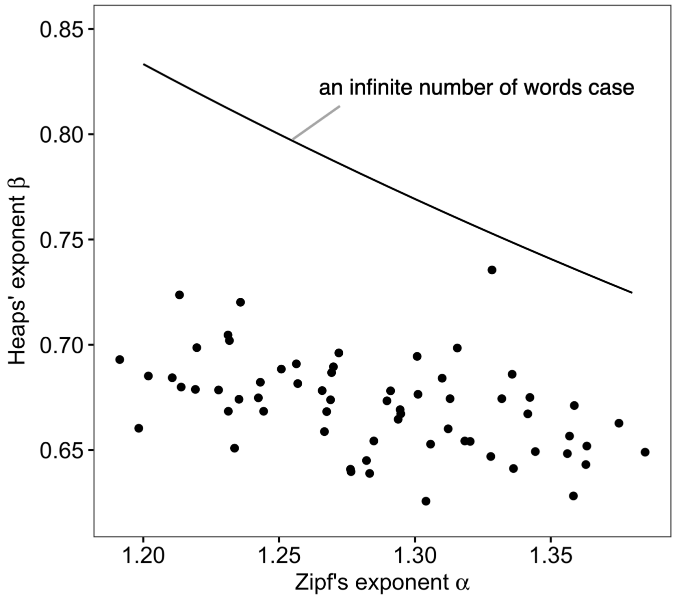

S5 Fig. Relationship between the estimated exponents in the case of no prefixes.

The prefixes were removed from words data. The correlation coefficient between α and β was −0.45

(p = 0.0002). Otherwise, same as Fig. 2 in the main text.

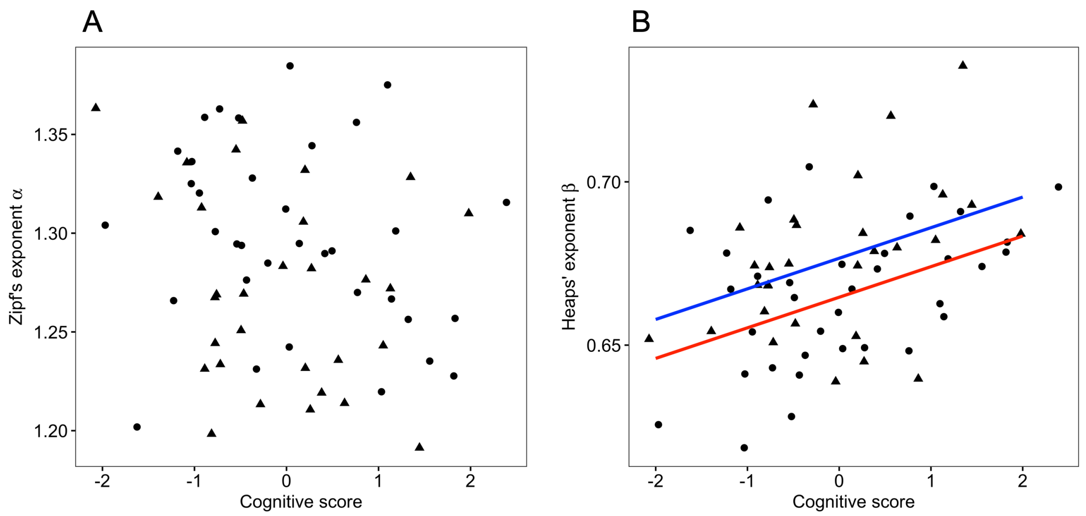

S6 Fig. Relationship between scaling laws and cognitive score in the case of no prefixes.

The prefixes were removed from words data. Otherwise, same as Fig. 3 in the main text.

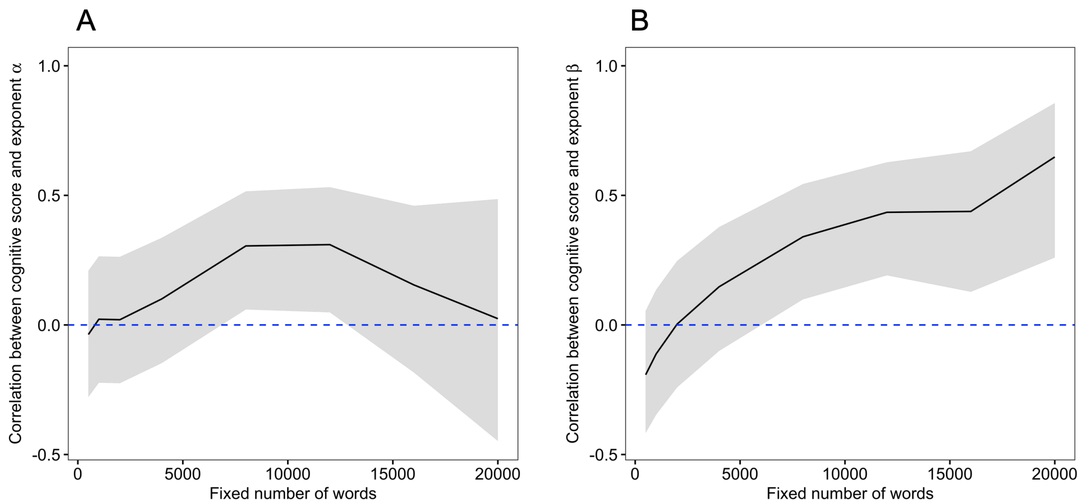

S7 Fig. Relationship between data length (fixed number of words) N_f and correlation coefficients with Zipf's and Heaps' exponents in the case of no prefixes.

The prefixes were removed from words data. Otherwise, same as Fig. 4 in the main text.

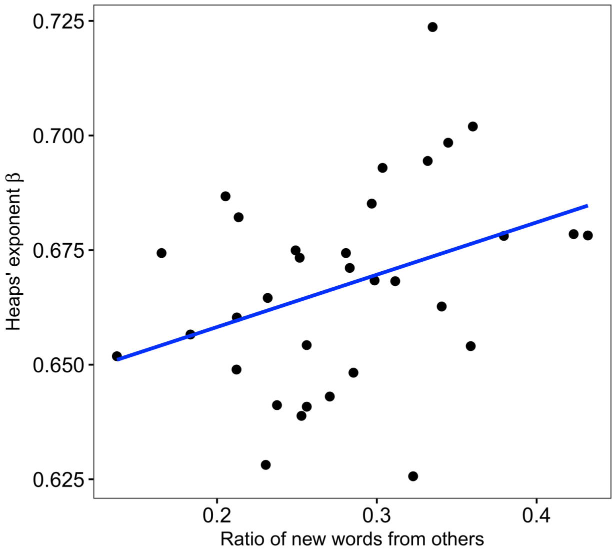

S8 Fig. Relationship between ratios of newly-used words from others and Heaps' exponents in the case of no prefixes.

The prefixes were removed from words data. The correlation coefficient between ratios of newly-used words from others and β was 0.36 (p = 0.04). Otherwise, same as Fig. 5 in the main text.

S2 Table. Summary of linear mixed model on scaling exponents in the case of no prefixes.

	**Explanatory variable: estimates (SE, p-value)**				
	Cognitive score	Gender (male)	Conversation type (presentation)	Age	Constant
Zipf's exponent α	−0.011 (0.006, 0.084)	−0.027* (0.01, 0.021)	−0.011 (0.014, 0.467)	−0.007 (0.006, 0.237)	1.304** (0.01, $<$ 0.001)
Heaps' exponent β	0.009** (0.003, 0.002)	0.012* (0.005, 0.03)	0.0009 (0.0055, 0.88)	0.00005 (0.003, 0.99)	0.664** (0.0043, $<$ 0.001)

The linear mixed model had a random effect as a conversation group. The results were derived from data sets without prefixes. (* $p < 0.05$, ** $p < 0.01$)

S3 Table. Regression coefficients of each raw cognitive score for the scaling exponent β in the case of no prefixes.

Cognitive score	Estimate (SE)	p-value
MoCA-J	0.0037 (0.001)	0.0007
Logical memory (I + II)	0.001 (0.0004)	0.017
Digit symbol coding	0.0005 (0.0002)	0.018
Digit span (forward + backward)	−0.0009 (0.001)	0.367

The linear mixed model had a random effect as a conversation group. The results were derived from data sets without prefixes.

References

1. Clauset, A., Shalizi, C.R., Newman, M.E.J., 2009. Power-Law Distributions in Empirical Data. SIAM Rev. 51, 661–703. https://doi.org/10.1137/070710111

2. Gerlach, M., Altmann, E.G., 2013. Stochastic Model for the Vocabulary Growth in Natural Languages. Phys. Rev. X 3, 021006. https://doi.org/10.1103/PhysRevX.3.021006

3. Burnham, K. P., Anderson, D. R., 2004. Multimodel inference: understanding AIC and BIC in model selection. *Sociol. Methods Res.* **33**(2), 261-304. https://doi.org/10.1177/0049124104268644
