# Supplementary material for: Scaling laws in natural conversations among elderly people
Source: PLoS One. 2021 Feb 19;16(2):e0246884. doi: 10.1371/journal.pone.0246884 (PMC7894956; doi:10.1371/journal.pone.0246884)
Supplement: Attachment — Submitted filename: Responses.pdf [file pone.0246884.s005.pdf]

Dear Editor:

Please consider the attached manuscript “Scaling laws in natural conversations among elderly people” for publication in PLOS ONE. The current material has not been published nor is it under consideration for publication elsewhere. The point-to-point responses to the reviewer’s valuable comments are enclosed in the submission. Thank you very much in advance for your consideration.

Dr. Masato S. Abe
RIKEN Center for Advanced Intelligence Projects,
Nihonbashi 1-chome Mitsui Building, 15th floor,
1-4-1 Nihonbashi, Chuo-ku, Tokyo 103-0027, Japan
Tel: +81-048-467-3627
masato.abe@riken.jp

Answers to Reviewer 1

In response to the valuable comments of the referee, I have modified my paper appropriately to address her/his concerns. Below I give a point-by-point reply to each criticism. Please note that the improved parts in the manuscript are indicated in blue.

Comment 1

Before that, I would like to discuss again the format of the paper. In this version that you have submitted, when a link to a graph is clicked, the focus moves to the caption of the graph, but the graph is somewhere else. It is extremly upsetting to read a paper like this. If in the previous version the problem was that you were using Word instead of latex, I do not see why this still happens when you use latex. As you may know, latex has a way to place the graphs in the pace where they are defined in the .tex file ([h!]). Still worse, the graphs are vertical instead of horizontal. That means that when I have a reference to your graph, I click it, and then, I just see a caption, and then, I need to find (and figure out) where the graph is and which one it is, and after all this, then, I need to rotate the page 90 degrees in order to be able to read it.
We review papers just as a service to the community, and not only for free, but also at the expenses of our efforts and time. This is acceptable, and I do not complain for it, I am happy to help, but I do not accept that I need to read the paper in such an uncomfortable and difficult way, specially when the tools to prevent this mess are easily available. This comment is not only for the authors, but for the editors as well.

Response: We are sorry for making you uncomfortable again. We understand what you mean. However, this is a PLOS ONE's problem. The position of the graphs must be at the end of the manuscript even for Latex format, according to the submission guidelines of PLOS ONE. The guideline also says that clicking the figure references in the text should take you to not the figures, but the caption. The Latex template of PLOS ONE is designed to do so. Thus, we followed these guidelines.

Comment 2

First of all, at the end of the abstract the authors state that: "We found that word patterns followed these scaling laws irrespective of cognitive function, and that the variations in Heaps' law were associated with cognitive function. Moreover, variations in Heaps' law were associated with the ratio of new words taken from the other participants' speech. These results indicate that scaling laws in language are related to cognitive processes.". This whole paragraph is contradictory. They say that "these scaling laws [are] irrespective of cognitive function", this is "these laws" are both Zipf's and Heaps', and then, they say: "variations in Heaps' law were associated with cognitive function". The following sentence still adds more contradiction to the sentence. In any case, I guess that this is just a writing error.

Response: Thank you for your comments. We considered two points about scaling laws: 1) whether the participants exhibit scaling laws and 2) if they exhibit scaling laws, is there a variation in exponents? And are these exponents related to cognitive functions? These raised problems are written in Introduction (lines 62-69).

For the first question, we analyzed the rank-frequency relationship of words and the number of words and different words relationship and evaluated the goodness-of-fit. We confirmed that all they commonly exhibited two scaling laws. Therefore, this point is not irrespective of cognitive

functions. For the second question, we found the variations in scaling exponents and the significant relationship between the exponents β and cognitive functions. Therefore, although whether words patterns follow scaling laws or not is irrespective of cognitive scores, the variations in the exponent β is associated with cognitive scores. To avoid confusion, we improved the abstract.

Comment 3

In line 53, the authors say that ”Heaps' law describes how new words are produced along with sentences or during conversations”. This is not the meaning of this law, and I think that the mistake on the interpretation of this law implies more serious problems along the paper. Heaps' law state that the number of different words in a text is a funtion proportional to the length of the text (modulo exponent). This has nothing to do with the idea of ”new” words, in the sense of words that did or did not belong to the speaker's lexicon. This law describes how the variety of different words varies when we write or speak. It may seem that the only problem of the interpretation of the meaning of this law is just that the authors use the word ”new” where I use the word ”different”, but I will discuss it later, to show that, from this reviewer's point of view, the authors have mistaken or misused the meaning of this law.

Response: Thank you for your comments. In our paper, we use ”new words” as newly-used words (or newly-appeared words) in each participant's data set. We do not mean that new words are added to his/her vocabulary or lexicon. Such a way to use ”new words” appeared in previous studies (e.g., Gerlach and Altmann 2013). Anyway, to avoid confusion, we removed the sentence in line 53 of the previous manuscript and changed ”new words” to ”newly-used words” or ”different words” in a whole manuscript.

Comment 4

My main concern with the results shown in the paper are related to the Heaps' law results.
In line 227 the authors state that:
”the relationship between the exponent β of Heaps' law and cognitive scores and found a significant relationship (p = 0.002)”.
But there is a value that goes along this p-value, which is 0.003. What is the meaning of this value? Or, said otherwise, the fact of being statistically significant is important, but then, we need to see the slope (in case of a correlation analysis) or an extra metric that describes the nature of this significant p-value. To make it clearer, when you have a significant correlation, then you have a look at the slope, since it is not the same to have a significant correlation of a slope -¿ 0 than a significant correlation with a slope -¿ 1/-1. In this case, apart from the significance, what else can be said about the nature of both relations? This is important to clarify because of Zipf's and Heaps' laws are connected, then, the authors need to be very precise when they state that the expected behavior (assuming transitivity between the relations: cognitive score - Zipf's law, cognitive score-Heaps law, Heaps' law - Zipf's law) does not hold according to they results.

Response: Table 2 shows the statistical result of the linear mixed model, including the slopes. ”Explanatory variable: estimates (SE, p-value)” in the upper of Table 2 indicates that values in each cell represent the estimated regression coefficient, the standard error of the coefficient, and the p-value. For example, the regression coefficient, the standard error, and the p-value in cognitive score vs β are 0.009, 0.003, and 0.002, respectively. The result indicates that as the cognitive scores increases by 1, beta increases by 0.009.

Also, there are inevitable random factors in the relationship between variables. Hence, tran-

sitivity does not always hold. We discussed the relationship among α, β, and cognitive functions (lines 297-306).

Comment 5

In lines 231-234 the authors state that: "We confirmed a robust relationship between the exponent β and all original cognitive scores, except for the digit span (Table 3)." (apart from the very liberal use of the word "robust" in this particular case) and then they say: "Thus, these results indicate that the variation in Heaps' law could be associated with the difference in cognitive functions." Yet, you also state that: 1. there is no relationship between cognitive score and number of uttered words (line 217). 2. in figure 4 you show a relationship between cognitive score and exponent β and length of text. This seems inconsistent, taking into account that transitivity should apply in these cases. I did not find any discussion about this fact in the paper.

Response: Thank you for your comments. We meant no significant correlation (calculated by Spearman's correlation coefficient) between the number of words and the cognitive function scores. To avoid confusion, we improved the sentence (line 217).

In Figure 4, the x-axis is the fixed word length, and the y-axis is the correlation coefficient between cognitive scores and β. It suggests that many words make the relationship between cognitive scores and β clear. Therefore, it does not indicate that participants with a large number of words have high cognitive scores. Thus, it is not related to the transitive relationship.

Comment 6

Finally, I would like to comment section "Source of new words". After the response to one of my questions, I firmly think that the definition of "new words" that they apply in this paper has nothing to do with the meaning of Heaps' law. What they do is to analyze the relation of different words only if they have been uttered before by another speaker. This is not what the law states, since the law makes no difference about the "origin" of the words or if they were already in the speaker's lexicon or they just learned it. This law measures the proportion of different words w.r.t. number of uttered words. Therefore, selecting only those words that have been uttered by someone else, the authors are biasing the analysis. I do not see any meaning on analyzing only this subset of words.

Moreover, in order to compute the parameter of Heaps' law, it seems clear that the longer the text is, the more accurate the computation of this parameter will be, since this function will have a larger size span to be fitted. And *precisely* because of that, this parameter *needs* to be computed with fixed length text, if the purpose of the analysis is to see relevant differences between speakers' performance. It comes to no surprise to me that the longer the text, the higher the value of this parameter is. In fact, I would say that the longer the text is, the more *accurate* the value of this parameter is (but this is just a guess).

If I had to see if this parameter had an impact or a relationship with the cognitive score, I would take some individuals with a significant low score and some with a significant high score (w.r.t. average, for instance), obtain their uttered words, set a prefix length fair for all of them (the minimum is usually taken), compute the Heaps' parameter for all of them, and then, apply a method to see if the difference (in average, for instance) is significant. Or you could group them as well (low, medium, high cognitive score), and see the statistical differences between all of them. Using you methodology, you make a mistake (from my point of view):

1. using the words that you define as "new". 2. not setting a prefix length. 3. not using more precise and clearer (and yet simple) statistical tools to find out the relation between individuals.

In is not enough to see if there is a relation between the cognitive score and the length, because not taking a prefix to compute Heaps' parameter is biased by you decision of taking a fixed prefix length.

In fact, the last graph may mean nothing, since you are not using all the available utterances for the analysis, and the graph B in the previous page may just mean that the more words you take, the more precise is your computation of the Heaps' parameter.

That means that your statement in lines 298-299 is dubious.

Response: Although you may misunderstand our analysis, we analyzed all available words in the conversation for scaling laws, not limited to words uttered before by another speaker. In the section of Source of new words, we analyzed the origin of newly-used words in each participant. It can be independent of scaling laws. Also, we already showed the result of the fixed-length data for scaling laws in Figure 4. As for prefix, we analyzed data without prefix. However, the results almost never changed (lines 234-236). We added them to Supporting Information (Appendix S3, S4-S8 Figs, and S2-S3 Tables). Additionally, we found a mistake in a caption of Fig. 5 and improved it.
